# A Non-Intrusive Monitoring System on Train Pantographs for the Maintenance of Overhead Contact Lines

**DOI:** 10.3390/s23187890

**Published:** 2023-09-14

**Authors:** Borja Rodríguez-Arana, Pablo Ciáurriz, Nere Gil-Negrete, Unai Alvarado

**Affiliations:** 1 Ceit-Basque Research and Technology Alliance (BRTA), Manuel Lardizabal 15, 20018 Donostia/San Sebastián, Spain; 2 Universidad de Navarra, Tecnun, Manuel Lardizabal 13, 20018 Donostia/San Sebastián, Spain

**Keywords:** pantograph–catenary interaction, infrastructure monitoring, railway, experimental results

## Abstract

The condition monitoring of an overhead contact line (OCL) is investigated by developing an innovative monitoring system for a pantograph on an electrical multiple unit of a regional line. Kinematic and dynamic modelling of the pantograph is conducted to support the designed monitoring system. The modelling is proved through rigorous test-rig experiments, while the proposed methodology is then validated through extensive field tests. The field tests serve a dual purpose: First, to validate the monitoring system using benchmark measurements of the tCat^®^ trolley, and second, to assess the reproducibility of measurements in a realistic case. This paper presents the OCL monitoring system developed in the framework of the H2020 project SIA. The accuracy of our results is not far from that of other commercial systems, with just 12 mm of absolute error in the height measurement. Therefore, they provide reliable information about trends in various key performance indicators (KPIs) that facilitates the early detection of failures and the diagnosis of anomalies. The results highlight the importance of model calibration and validation in enabling novel health monitoring capabilities for the pantograph. By continuously monitoring the parameters and tracking their degradation trends, our approach allows for optimized scheduling of maintenance tasks for the OCL.

## 1. Introduction

**Figure 1:**
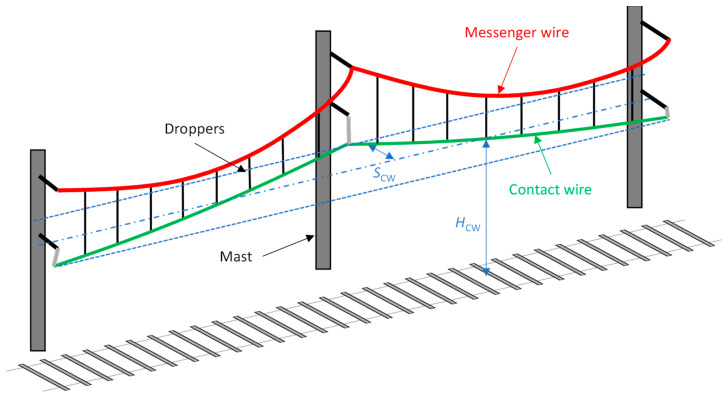
Schematic representation of the overhead contact line (OCL).

The overhead contact line (OCL) or catenary system is a key element of the railway infrastructure that provides the supply of energy to the electrical vehicles running through a line. The electric current is transferred from the contact wire (CW) to the vehicle through pantographs installed on the roof. The pantograph and OCL interaction depends on their mechanical characteristics [1] and contributes to the degradation of both systems with regard to their design configurations. Monitoring such interactions allows a good characterization of the health status of the OCL. Furthermore, maintenance operations are scheduled periodically to recover the original state of the OCL based on the data gathered from periodic (however scarce) inspections. These data are normally provided by expensive equipment installed in dedicated vehicles and processed by expert data analysts. However, this maintenance approach increases the life cycle cost (LCC) of the railway infrastructure. A preventive opportunistic maintenance strategy can reduce the number of maintenance schedules [2]; the maintenance of an OCL could, therefore, be optimised by monitoring some of their characteristics, such as the height and stagger of the CW (Figure 1), from sensors placed on pantographs and reducing the LCC. In addition to maintenance, the prediction of probabilistic risks is also being studied [3].

The geometrical study of the pantograph frame is described by Benet et al. [4], considering a three-dimensional model. Generally, the static force at the head of a pantograph remains constant regardless of the working height. This can be achieved through the accurate design of the geometry and the mechanism to lift the pantograph [5]. Not only the static lift force but also the tension of the CW have a significant influence on the dynamic performance of the pantograph and catenary system [6]. The deviation of the dropper length and the mast height from their nominal values is primarily responsible for tension changes [7]. Vesali et al. proposed a fast and accurate method for determining the static equilibrium configuration of a catenary [8]. Regarding the dynamic behaviour, wind loads and the irregularities of the CW also have a significant influence [9,10]. The dynamic performance due to local dropper defects has also been studied [11]. Most of the models employed are gathered together in the benchmark presented by Bruni et al. [12]. In these models, pantographs are usually modelled by two or three lumped masses, whose parameters can be obtained through experimental testing [13]. Accurate pantograph–catenary models provide a suitable approach for different investigations, such as assessing the current collection quality [14] or monitoring the stagger [15]. In addition to models, test facilities are developed to study the interaction between pantographs and OCL systems [16].

Inspection and monitoring are closely related concepts associated with railway infrastructure maintenance. Inspection entails systematic examination of the condition of different railway components, such as tracks, OCL, bridges, tunnels, and other structures, utilising sensors, cameras, and similar technologies. Typically conducted at regular intervals, such as annually or bi-annually, inspections aim to identify potential issues and defects that need to be repaired. On the other hand, monitoring involves the continual or regular gathering of data on the condition of railway components. These data are subsequently analysed to detect trends and patterns that may imply potential problems or concerns. The primary objective of monitoring is to enable early detection of potential issues and facilitate timely intervention before they escalate into major problems. Both inspection and monitoring serve as crucial instruments for ensuring the safety and reliability of the railway network. While inspections provide a detailed snapshot of the condition of railway components, monitoring allows a more continuous and real-time evaluation of their state.

The deployment and installation of infrastructure monitoring systems can vary, leading to different approaches [17]. Wayside monitoring and on-board monitoring are two distinct methods employed in assessing railway infrastructure. Wayside monitoring involves the utilization of sensors and stationary technologies positioned alongside the railway tracks, such as on the ground, on bridges, or within tunnels. These technologies gather data on the condition of vehicles and some network assets. Conversely, on-board monitoring entails the incorporation of sensors and embedded technologies directly on trains. These technologies collect data on the condition of the trains, their components, and the railway infrastructure’s assets as the train traverses over them. On-board monitoring facilitates the real-time collection of data on the railway network’s condition, enabling the identification of potential issues and defects in linear infrastructure, such as the OCL. In this case, different technologies can be used to assess the health status of the railway OCL. These technologies enable the detection of deformations such as sagging, twisting, or other anomalies that could impact the performance of the OCL system. Some examples of these technologies include laser scanners, which use lasers to precisely measure the position and alignment of the CW [18]; infrared cameras, capable of detecting temperature variations and other anomalies within the OCL system [19], aiding in identifying potential issues; other vision-based technologies for anomaly detection and failure diagnosis in the catenary equipment [20]; accelerometers, which measure the vibration of the wires and other related structures, providing valuable insights into their dynamic behaviour [21]; and distributed acoustic sensing (DAS) systems, which use fibre optic cables along the lines or in the pantograph to detect defects along the OCL system and its components [22].

The above-mentioned monitoring systems for OCL can pose challenges in terms of cost, installation, and maintenance, placing a significant burden on railway operators. To tackle this issue, there is an increasing demand for low-cost monitoring systems that offer ease of installation and maintenance, without the need for specialized tools or expertise. These systems should also have the capability to gather detailed and precise data regarding the condition of the OCL system. This enables early detection of potential issues and failures, facilitating timely repairs and maintenance interventions. By addressing the cost and complexity associated with monitoring, these low-cost systems aim to alleviate the burden on railway operators and enhance the overall efficiency of OCL maintenance.

Therefore, this work aims to develop a non-intrusive and low-cost monitoring system for an OCL that leverages the modelling of the pantograph. The system uses affordable MEMS (micro-electro-mechanical systems) sensors in the range of a few dollars, significantly reducing costs (up to 2–3 orders of magnitude) compared to traditional railway-specific and optic-based systems. While our system does not cover all the parameters covered by the EN50317 standard [23], it offers a crucial set of key performance indicators (KPIs). These KPIs include geometrical parameters (CW height and stagger) and an estimation of the static contact force, ensuring a comprehensive evaluation of the OCL condition. Initially, the modelling of a pantograph is implemented in MATLAB to consider the kinematics and static loads within the working range of the panhead height. Subsequently, experimental tests are conducted on a test rig to evaluate the actual characteristics of the pantograph. Based on the findings from these prior tasks, the design of a non-intrusive monitoring system is developed. Finally, the effectiveness of the developed system is validated through field tests, which yield results comparable to those obtained through conventional measuring methods.

## 2. System Modelling

In the current research, some models for simulating the mechanical behaviour of a pantograph are proposed, which enable the simulation of various pantograph details while accommodating changes in the characteristics of its components. The models have been successfully implemented using MATLAB and provide interesting results regarding contact height, static contact force and stagger of the CW. The proposed models are valid for any pantograph, but note that the torque calculation employed for the static contact force model is only for this specific design.

The model adopted for the kinematical assessment considers the structure of the pantograph to be a one-degree-of-freedom articulated quadrilateral mechanism. The symbolic solver of MATLAB allows calculating the different positions of the assembled structure, which comprises three primary parts: the push bar (*a*), lower arm (*c*) and connections on the upper arm (*b*), given an inclination value of the lower arm according to

(1) a·cos(α−φ)+b·cos(β−φ)−c·cos(γ−φ)−d=0,

(2) a·sin(α−φ)+b·sin(β−φ)−c·sin(γ−φ)=0,

where:

- a, b, c are the length of the three bars of the articulated quadrilateral mechanism.
- α, β, γ are the inclination against the horizontal of a, b and c bars.
- d is the distance between fastening points.
- φ corresponds to the angle of fastening points against horizontal.

**Figure 2:**
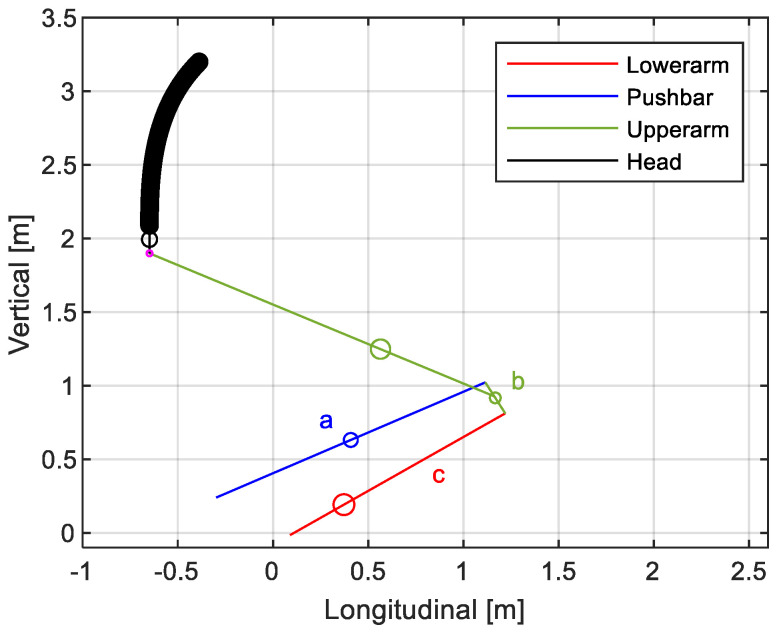
Simplified representation of a pantograph model and movement of the pantograph head.

The solution obtained from the articulated quadrilateral mechanism defines the entire upper arm part of the pantograph. Figure 2 shows the downward movement of the pantograph head, where the head trajectory is defined by the dimensional characteristics of the structure. The parameters used in the articulated quadrangle are summarized in Table 1. The upper arm bar connected to b has a length of 2.055 m and a relative inclination of 35.3 degrees.

**Figure 3:**
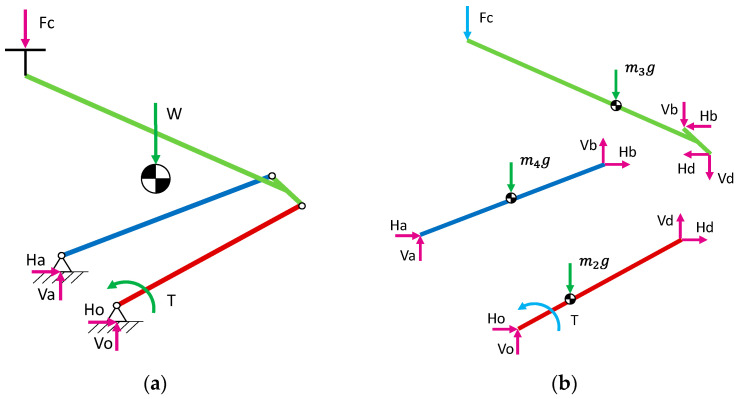
Simplified representation of a pantograph model. (**a**) Assembled and (**b**) breakdown structure.

The uplift force of the pantograph originates from a torque applied to the lower arm. When the strips make contact with the CW, the pantograph reaches a steady state. This steady state can be evaluated for each position in a 2D analysis. The maximum vertical slope of a CW should be 2‰. Therefore, at the maximum speed of a regional EMU train (around 25 m/s), the vertical speed of the panhead could reach less than 0.1 m/s and dynamic effects on pantograph arms can be avoided. The supports for both the lower arm and the push bar are constrained in the vertical and longitudinal directions. As a result, the structure of the pantograph becomes hyperstatic, and a breakdown of its components is required to solve the problem. Figure 3 illustrates the unassembled structure, which facilitates the definition of a system comprising nine equations and nine unknowns, as follows:

(3) $$\left(\begin{array}{ccccccccc} 1 & 0 & 1 & 0 & 0 & 0 & 0 & 0 & 0\\ 0 & 1 & 0 & 1 & 0 & 0 & 0 & 0 & 0\\ a_{31} & a_{32} & 0 & 0 & 0 & 0 & 0 & 0 & 0\\ 0 & 0 & 0 & 0 & 1 & 0 & 1 & 0 & 0\\ 0 & 0 & 0 & 0 & 0 & 1 & 0 & 1 & 0\\ 0 & 0 & 0 & 0 & a_{65} & a_{66} & 0 & 0 & 0\\ 0 & 0 & -1 & 0 & 0 & 0 & -1 & 0 & 0\\ 0 & 0 & 0 & -1 & 0 & 0 & 0 & -1 & -1\\ 0 & 0 & 0 & 0 & 0 & 0 & a_{97} & a_{98} & a_{99} \end{array}\right)\left\{\begin{array}{c} H_{o}\\ V_{o}\\ H_{d}\\ V_{d}\\ H_{a}\\ V_{a}\\ H_{b}\\ V_{b}\\ F \end{array}\right\}=\left\{\bar{b_{F}}\right\},$$

(4) $$\left(\begin{array}{ccccccccc} 1 & 0 & 1 & 0 & 0 & 0 & 0 & 0 & 0\\ 0 & 1 & 0 & 1 & 0 & 0 & 0 & 0 & 0\\ a_{31} & a_{32} & 0 & 0 & 0 & 0 & 0 & 0 & a_{39}\\ 0 & 0 & 0 & 0 & 1 & 0 & 1 & 0 & 0\\ 0 & 0 & 0 & 0 & 0 & 1 & 0 & 1 & 0\\ 0 & 0 & 0 & 0 & a_{65} & a_{66} & 0 & 0 & a_{69}\\ 0 & 0 & -1 & 0 & 0 & 0 & -1 & 0 & 0\\ 0 & 0 & 0 & -1 & 0 & 0 & 0 & -1 & 0\\ 0 & 0 & 0 & 0 & 0 & 0 & a_{97} & a_{98} & 0 \end{array}\right)\left\{\begin{array}{c} H_{o}\\ V_{o}\\ H_{d}\\ V_{d}\\ H_{a}\\ V_{a}\\ H_{b}\\ V_{b}\\ T \end{array}\right\}=\left\{\bar{b_{T}}\right\}.$$

where generally $a_{ij}$ terms depend on the geometrical design (distance units) of the structure and must be properly defined to obtain an equilibrium of forces and moments. The $a_{39}$ and $a_{69}$ non-dimensional terms, which are multiplied by a torque T, take 1 or 0 value as a function of where the torque is applied (joint a or o). The vectors $\bar{b_{F}}$ and $\bar{b_{T}}$ refer to the terms that are necessary to solve the contact force $F_{c}$ or applied torque T respectively. These terms depend not only on the geometrical design but also on the mass of each bar. The terms $H_{o,d,a,b}$ and $V_{o,d,a,b}$ solve the horizontal and vertical reactions at the ends of the bars, while neglecting any bending effects. Hence, the terms of $\bar{b_{F}}$ and $\bar{b_{T}}$ vectors meet the equilibrium of force in the horizontal direction (mainly zero), the vertical direction (mainly mass of the bar multiplied by gravity, with force units) and torque (depending on mass, forces on the ends and their distance from the assessed point), respectively, for each bar of the structure.

As viscous and dry friction phenomena in articulated and prismatic joints are neglected in the model for the estimation of the static contact force, the calculated contact force for a known height should be given along with a confidence interval that can be obtained with previous tests.

The torque application mechanism on the lower arm can vary depending on the type of pantograph. In the case of the monitoring system here presented, the pantograph (of a regional train) is equipped with a passive torque actuator comprising two linear springs. However, the relationship between the applied torque and the inclination angle of the lower arm is non-linear. To achieve this non-linear torque characteristic, the pantograph incorporates a mechanical system that controls the distance between the spring and the rotation centre of the lower arm. This control is accomplished using two screws, which determine the position of a one-degree-of-freedom mechanism consisting of a helical spring and three bars.

The helical spring is elongated from its nominal length within the working range of the pantograph structure, exerting a force $F_{s}$. The applied torque T, opposing the rotation of the lower arm (denoted by γ), can be calculated using the stiffness of the linear spring $K_{s}$, its elongation $\delta_{s}$, and the distance to the rotation centre of the lower arm $d_{s}$, instead of a non-linear rotational stiffness $K_{\gamma }$ and the accumulated rotation, according to

(5) $$T\left(\gamma \right)=K_{\gamma }\cdot \gamma =F_{s}\cdot d_{s}=K_{s}\cdot \delta_{s}\cdot d_{s}.$$

**Figure 4:**
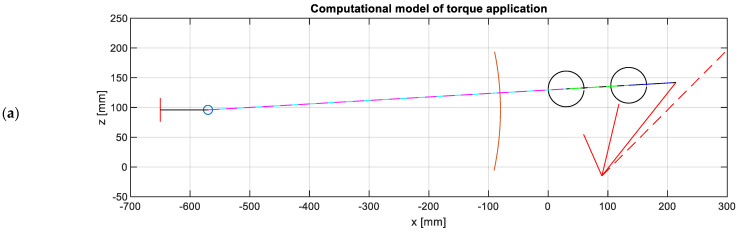

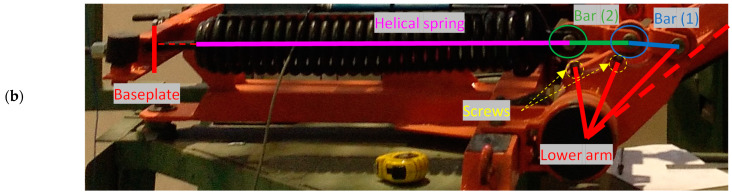
(**a**) Computational modelling of torque application and (**b**) schematic representation above the real mechanism.

The mechanical system that introduces a non-linear torque to the pantograph structure has been modelled using MATLAB. This mechanism consists primarily of four bars, one of which (associated with helical springs) can change its length and is joined at one end to the baseplate. Another bar is fixed to the lower arm and the screws that control the mechanism, ensuring that their relative rotation with respect to the rotation centre is the same. The remaining two bars have circular-shaped ends, and their relative movement with the preceding bar becomes negligible once contact with the corresponding screw is established. Considering the lowering of the pantograph structure, before contact occurs, the bars with circular-shaped ends and the changeable bar are aligned. Figure 4a shows the representation of the computational model, where the elongation $\delta_{s}$ and distance $d_{s}$ are calculated for each inclination of the lower arm. Figure 4b provides a schematic representation of this model, overlaid on the actual mechanism.

Once the kinematical assessment of the mechanism of torque application is completed, the applied torque, as a function of the inclination of the lower arm, T(γ), is found to be proportional to the stiffness of the helical spring. Using Equations (3) and (4), the static contact force $F_{c}$ on the top of the pantograph structure can be determined. Thus, the geometrical characteristics, the mass of the pantograph structure, the spring stiffness, and the distance of screws are sufficient to calculate the static contact force.

The system modelling approach employed in this study for the stagger assessment is based on the signal processing techniques used in the OCL model developed by Blanco et al. [15]. This model has a lumped mass representation of the pantograph, allowing for its interaction with a 2D OCL model. The model calculates panhead accelerations that, after SAWP signal processing, enable the monitoring of stagger in OCL systems. The calculation of the static force applied at the lowest mass of the lumped mass model, aimed at maintaining the contact force close to its nominal value, follows the guidelines outlined in the standard EN-50367 [24]. To ensure accurate results from this model, the lumped mass model should be representative of the pantograph installed on the train roof. For that purpose, instead of relying on parameter estimation from real tests, a linear system analysis (LSA) utilising multibody systems (MBS) can be employed [25].

In the system proposed here, the accelerations are directly obtained from sensors on the panhead, and only SAWP signal processing is employed without the need to use the dynamic model in [15].

## 3. Test Rig Experiments

**Figure 5:**
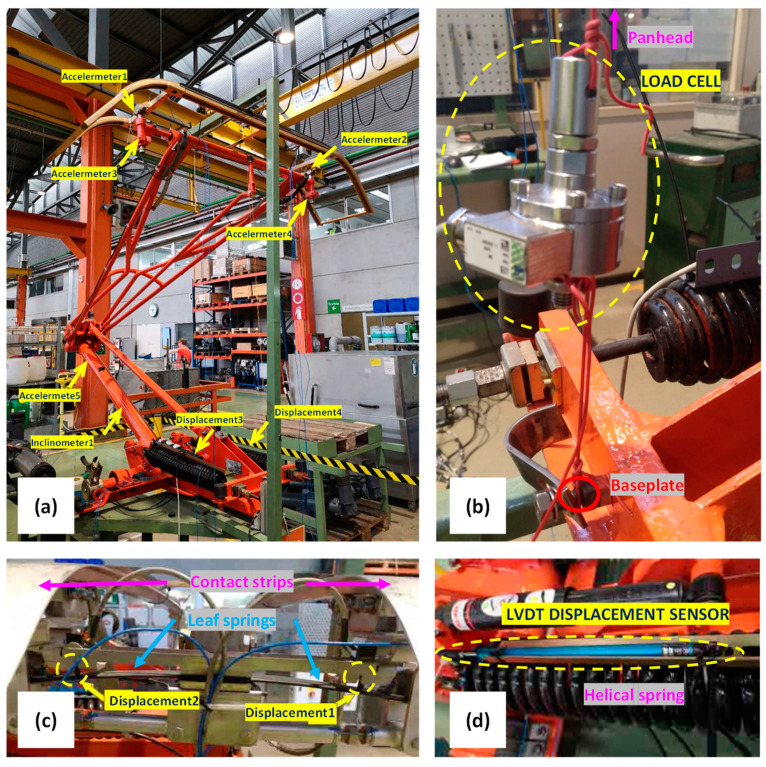
Pantograph instrumentation for test-rig experiments: (**a**) overall placement of accelerometers and displacement sensors; (**b**) load cell for the steady-state force at panhead; (**c**) displacement sensors on panhead leaf-springs and (**d**) displacement sensor for helical springs of structure.

To validate the developed model, test-rig experiments were conducted using an actual pantograph. The tests were carried out at the facilities of the Spanish train operating company FGC (Ferrocarrils de la Generalitat de Catalunya) on a working table designed for the calibration of pantographs. During these experiments, multiple acceleration and displacement sensors were strategically placed on the pantograph. An inclinometer was mounted on the lower arm to serve as a reference for the position of the structure. Furthermore, a load cell was utilised to measure the force at the panhead for different height positions. The configuration of the sensors on the pantograph is depicted in Figure 5.

The panhead of the pantograph was positioned at different heights within the operational range, from 0.5 to 2.5 metres. For each position, three measurements were considered:

- Shaft distance from baseplate
- Rotation angle of the lower arm from the horizontal
- Deformation of the helical spring relative to a previous reference.

**Figure 6:**
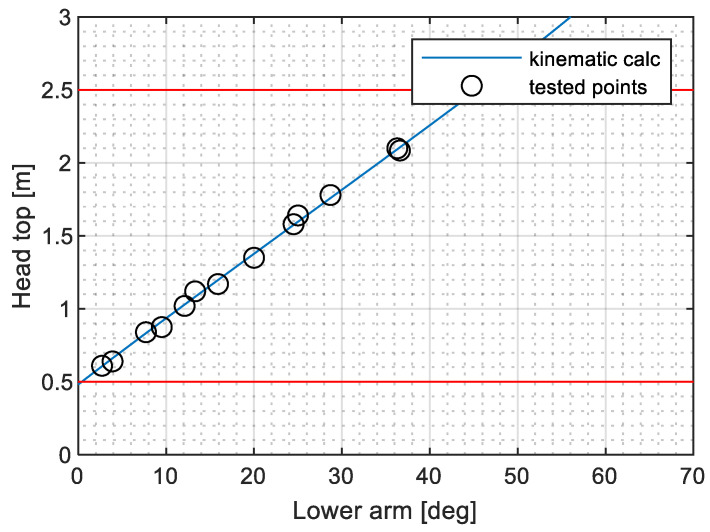
Comparison between measurements and kinematical assessment of the structure.

**Figure 7:**
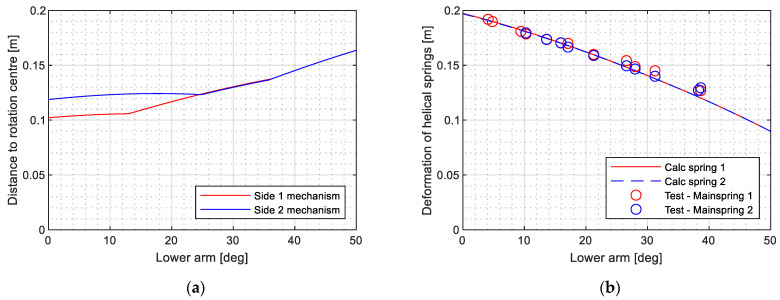
Computational assessment of the torque mechanism by (**a**) distance of springs to rotation centre and (**b**) their deformation comparing with test results.

Figure 6 presents a comparison between the measured shaft height and the inclination of the lower arm, as well as the kinematic assessment of the pantograph structure. The obtained results confirm the accuracy of the dimensions of the bars used in the system model and their links to the base frame. The computational assessment of the torque application mechanism provides the deformation of the helical springs and their distance to the articulation point between the lower arm and base frame. Figure 7a shows good agreement with the measured relative displacements. Figure 7b provides the distance obtained by simulation, which could not be directly measured but aligns well with the expected behaviour.

**Figure 8:**
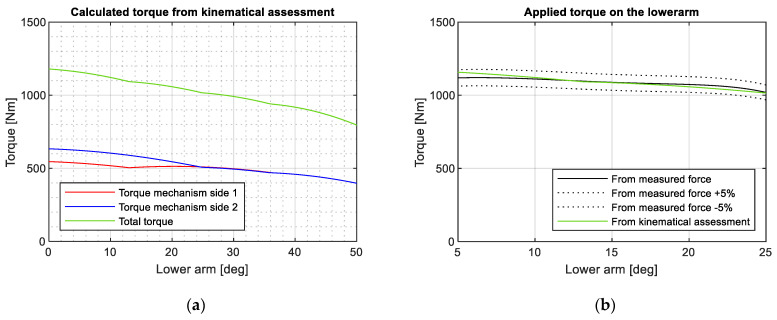
(**a**) Calculation of applied torque from kinematical assessment and (**b**) comparison with calculated torque from the measured force at shaft.

The deformation of helical springs and their distance to the articulated point enables the calculation of the non-linear torque provided by each spring. The total applied torque is obtained by summing the contributions from both mechanisms on each side of the pantograph, as depicted in Figure 8a. To validate the torque obtained from the kinematic assessment, a load cell is used to measure the force at different heights of the pantograph structure. By solving the system of nine equations and nine unknowns described in Section 2, it becomes possible to calculate the required torque for a known contact force at the panhead. Figure 8b illustrates that the torque calculated based on the kinematical assessment exhibits less than a 5% error compared to the torque derived from the measured force.

**Figure 9:**
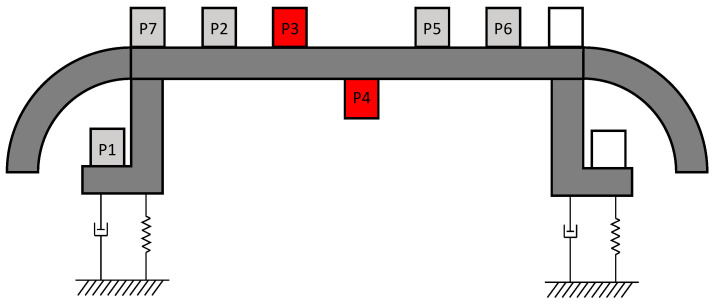
Sketch of accelerometers placed on the tested contact strip.

Additionally, to validate the model, some extra tests were conducted in order to characterise the dynamic behaviour of the contact strips of the pantograph. The modal shapes of these elements were determined using a dynamometric hammer equipped with a dynamic force sensor (DYTRAN 1051V4). To perform this analysis, seven accelerometers were strategically positioned on one of the contact strips of the pantograph’s head, as depicted in Figure 9. The structure of the pantograph was set in the lowest position and fixed to neglect additional movements. During the test, the strip was impacted vertically at the central position using the dynamometric hammer. A total of 5 impacts were recorded using a trigger for applied load and a time window of 5 s with a sampling rate of 800 Hz.

**Figure 10:**
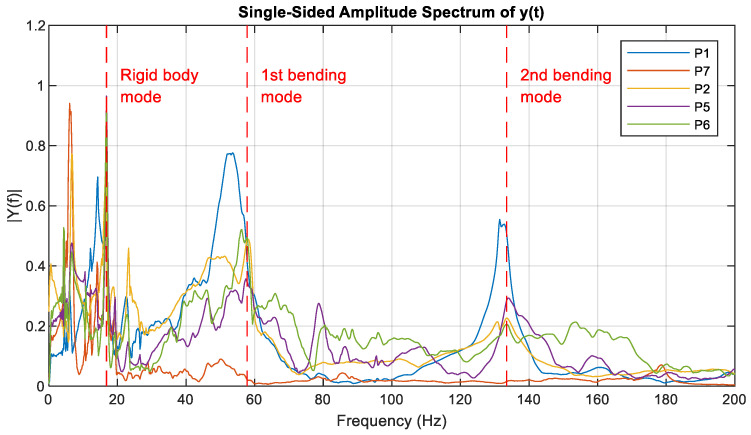
Averaged FFTs of measured points from five impacts with a time window of three seconds.

Afterwards, the recorded acceleration signals were post-processed using fast Fourier transforms (FFTs). Each signal is subjected to FFT analysis using a 3-s time window per impact and is normalised to its maximum value. It was necessary to discard the signals from channels corresponding to points P3 and P4 (coloured in red in Figure 9) due to acquisition errors. Figure 10 illustrates the resulting FFT for each point, representing the average of all impacts. Based on the identified peaks, the first and second bending modes are found to occur at frequencies of 57.78 Hz and 133.49 Hz, respectively. Additionally, the vertical mode of the rigid body can be observed at lower frequencies. It arises from the mass of the contact strip and the stiffness associated with the connection to the shaft of the pantograph structure.

Table 2 shows the frequency obtained experimentally for each vibration mode. These values are similar to those in the literature [26]. The results obtained could be used for the implementation of the structural modes of both lead and rear strips on an OCL model.

## 4. Monitoring System

Based on conclusions extracted from simulations, a low-cost and non-invasive monitoring system has been developed, focusing on the dynamic interaction of the pantograph and OCL (in operational service trains). The developed system aims to monitor the necessary information to generate different KPIs, i.e., CW height, the estimation of the contact force and the CW stagger. The design of the system adheres to two primary constraints. Firstly, the system’s hardware costs should be minimized to ensure affordability; a low-cost system is sought. Secondly, the installation process should be designed to be easy and non-invasive. These characteristics are crucial in enabling the monitoring system’s suitability for use in service vehicles during commercial operations. Nevertheless, as a counterpart, the quality and accuracy of the data obtained may be lower compared to more sophisticated systems that require complex installation (e.g., disassembling the pantograph’s head to accommodate force sensors to measure the contact force). The advantage of simpler and low-cost systems lies in their potential for installation in all trains operating within a network, which facilitates the generation of more frequent and pervasive data and offers additional value through further data analytics.

Based on that rationale, the developed monitoring system comprises several modules, namely sensors, positioning, processing and storage, communications, and power management. Each module contributes to the overall functionality of the system, ensuring efficient data collection, analysis, and transmission, while optimising power usage.

**Figure 11:**
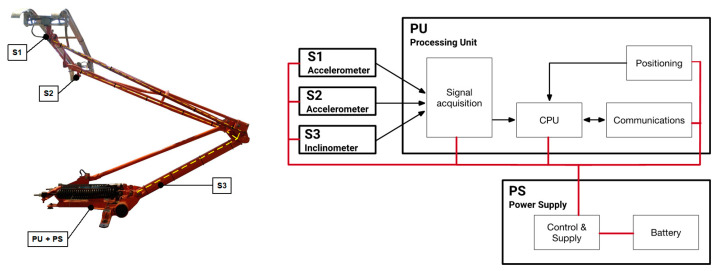
Architecture of the monitoring system and location of the different components in the pantograph.

The analysis described in the preceding sections has allowed the identification of the most suitable sensors for capturing the required signals, which will be further processed to generate the desired KPIs. The system includes two piezoresistive accelerometers (with a range of ±3 g and a bandwidth spanning from 0 to 500 Hz), mounted at both ends of the pantograph’s head. Furthermore, a dual-axis inclinometer/accelerometer (±90° and ±1.7 g) is placed at the lower arm (Figure 11).

Additionally, the monitoring system is equipped with a positioning module, which is necessary to generate both time and position stamps that are associated with the captured data. This module is based on a GNSS receiver that combines multi-constellation features (GPS and GALILEO) to enhance accuracy and availability. Providing an accurate (and available) position is key to geo-positioning the monitoring data of linear assets, enabling repeatability in the collected data. There are two key benefits to this approach. Firstly, it enables the precise localisation of defects along the railway line, facilitating the work of the maintenance staff in identifying and addressing issues. Secondly, when multiple trains provide large datasets that are accurately geo-positioned on the same line over an extended period (e.g., months or even years), it allows an advanced analysis to gain a better understanding of the health condition of the assets. This includes observing the evolution of various parameters over time at specific locations and contributing to comprehensive asset management and maintenance strategies.

The signals captured by the sensors are synchronised (time and position stamped) and processed onboard. The first step of the processing involves analysing the panhead accelerometries in order to detect impacts and shocks. When one of these events is detected, a real-time message is sent to a back-office application using an LTE wireless link. This message includes the severity and geo-localisation of the event, as well as additional contextual data such as train and service information. To facilitate the communication process, a publisher/subscriber system is implemented using an MQTT (message queuing telemetry transport) broker. The broker is subscribed to the events generated by the onboard monitoring system. Simultaneously, the raw data are processed to extract the target KPIs. The relevant information is then stored in binary files. These files are transferred via FTP (file transfer protocol). This transfer is carried out through the Wi-Fi installed in the stations. This approach ensures that there is sufficient bandwidth to transmit the files and minimises the risk of signal loss during the transmission process.

The power required to operate the electronics of the monitoring system is provided by a 12VDC GEL rechargeable battery. The negative terminal of the battery is connected to the pantograph structure and used as a reference voltage (i.e., virtual common ground) for the system, ensuring electrical isolation of the bodywork. To save energy during the pilot tests, a location-based trigger (using GNSS data) was implemented. This way, the recording of data can be controlled automatically and can be accessed and configured remotely. The architecture of the system and the placement of the different modules in the pantograph are depicted in Figure 11.

**Figure 12:**
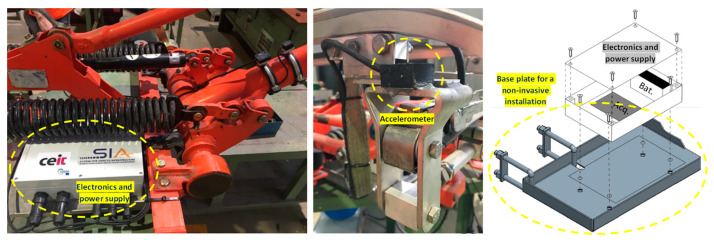
Installation of the monitoring system in the pantograph. Electronics and power supply (**left**); detail of an accelerometer (**centre**); detail of the base plate for a non-invasive installation (**right**).

The installation of the monitoring system involves securely attaching the three sensors to the pantograph’s structure using double-sided industrial tape. Prior to attaching the tape, cyanoacrylate-based glue is applied to the base of the sensor enclosure to ensure a firm and reliable bond. This installation method is non-invasive and does not hinder the normal functionality and free movement of the pantograph. The remaining electronic components and power supply share the same enclosure, which is mounted on the base frame of the pantograph using a specially designed base plate. The design is such that it does not interfere with the moving parts of the pantograph while maintaining proper clearance with the roof of the vehicle. The plate is attached to the frame using U-shaped metallic brackets, providing stability to the entire system. The sensor cables are neatly fixed and secured to the structure of the pantograph using zip ties. To accommodate the movement of the pantograph, sufficient space is provided for the cables to pass through any moving parts while adhering to the minimum flexion radius specified by relevant standards. Figure 12 provides details about the installation of the monitoring system in the pantograph.

Finally, regarding the results provided by the presented system, Table 3 lists a comparison with previous work where the employed methods are indicated.

## 5. Results of the Proposed System in Field Tests

This section focuses on assessing the measuring system through field tests conducted in real-world conditions. The accuracy and repeatability of the measuring system are crucial to ensuring the reliability and quality of the collected data. The results obtained from the field tests provide valuable insights regarding the ability of the system to accurately measure and record data, thus confirming its suitability for practical applications. The following subsections provide details about the case study conducted and present the significant results obtained, which contribute to the validation of the measuring system.

### 5.1. Description of the Case Study

The monitoring system was installed in a pantograph of a train by the Spanish train operating company FGC during regular service in commercial operations. FGC, founded in 1979, features more than 92 million passengers per year and also provides freight transportation services. With a dedicated workforce of more than 1900 people and an extensive network spanning 290 km of track with international, narrow and Iberian gauges, FGC operates more than 1300 train circulations per day, with a peak headway of 150 s on the Barcelona–Vallès line.

**Figure 13:**
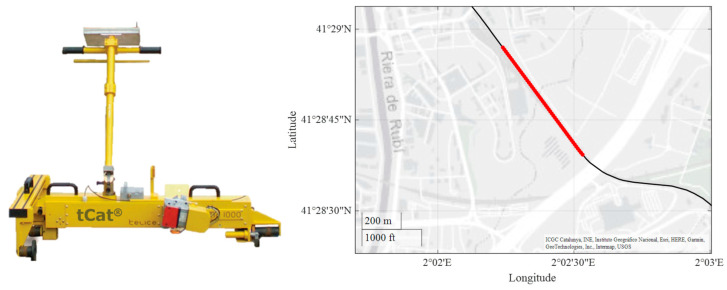
tCat^®^ measuring system (**left**) and track section used in the baseline validation campaign (**right**).

To establish a baseline for the condition of the OCL, an initial testing campaign was conducted using the tCat^®^ system [31], more precisely the tCat^®^ 1435 system. This system consists of a measurement stroller pulled by an operator that collects several lines of information about the track and the OCL. The measurements were carried out in track 2, specifically between the stations of Rubí and Hospital General (milestones 20 + 124 and 18 + 377, respectively, from Plaza Catalunya station reference), as depicted in Figure 13. The height and stagger of the CW in static conditions were recorded at all poles along the track.

To validate the results obtained from the developed monitoring system and compare them with the measurements taken with the tCat^®^, the proposed monitoring system was installed in a regular pantograph mounted on a unit of the 112 series while running through the same section as the tCat^®^. The results of this validation stage against the baseline measurements are shown in Section 5.2.1.

Furthermore, to ensure the repeatability of the measurements and gather more data for analysis, an additional measurement campaign was conducted. During this campaign, two different sections of the line were analysed, with two circulations performed in each section. Each section had a length of approximately 500 m. To save energy during regular operations, the collection of data was automatically triggered using the GNSS subsystem.

**Figure 14:**
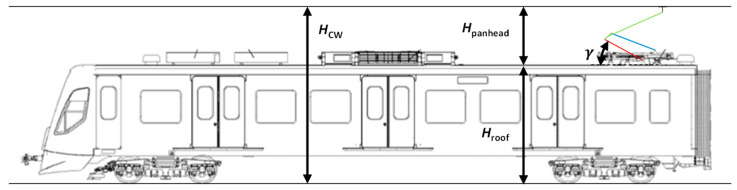
Scheme showing a graphical definition of important parameters of the height of the pantograph and CW.

Four parameters are analysed in each case: The stagger of the CW, the height of the pantograph, the height of the CW, and the estimated contact force. The contact force is estimated based on the experimental results from Section 3, particularly referring to Figure 8. The two height parameters are directly related and can be derived using the roof height. These relationships are graphically provided in Figure 14. The panhead height, and consequently the CW height, can be easily calculated using the kinematics of the mechanism and the angle of the lower arm (γ), as described by Equations (6) and (7). The parameters of these equations are obtained through linear fitting, as shown in Figure 6, and for a roof height of 3720 mm.

(6) $$H_{\mathrm{panhead}}\left(\gamma \right)\;\left[\mathrm{mm}\right]=493.953+\left(\gamma \cdot 42.877\right),$$

(7) $$H_{\mathrm{CW}}\left(\gamma \right)\;\left[\mathrm{mm}\right]=3720+H_{\mathrm{panhead}}\left(\gamma \right).$$

### 5.2. Results

This section presents the results obtained from the field tests. The results were validated using a commercial measuring device (tCat^®^), and subsequently, their repeatability was studied through multiple runs in different sections of the track.

#### 5.2.1. Comparison with Commercial Measurement Device

**Figure 15:**
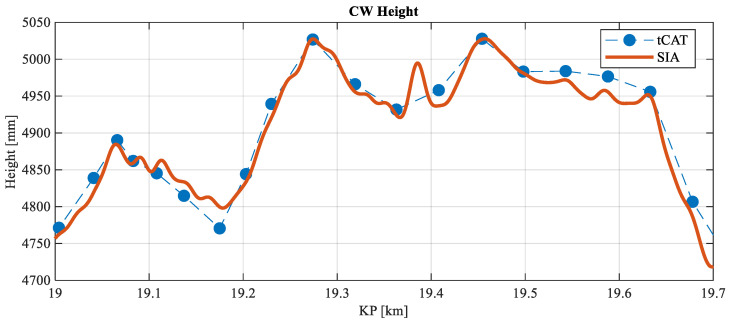
CW height comparison between tCat^®^ and the monitoring system.

**Figure 16:**
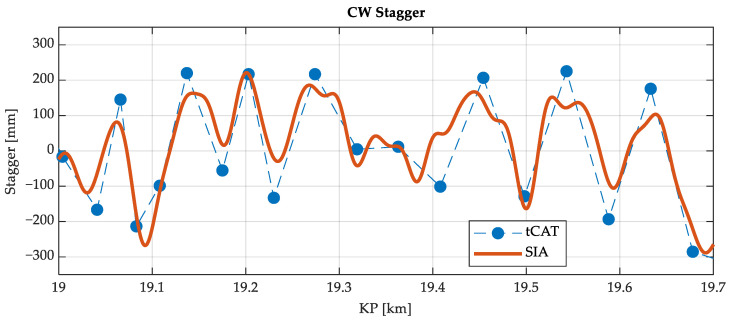
Calculated stagger with the monitoring system versus the reference values measured with the tCat^®^ system.

The initial analysis seeks validation of the measurements taken by the system by comparing them with the tCat^®^ commercial measurement system. Figure 15 presents the comparison of the CW height calculated by the monitoring system versus the reference values taken by the tCat^®^. In Figure 16, the results of the stagger of the CW are shown. Overall, the results show a very good correlation in the direct CW height measurements (Figure 15) and promising similarities in the CW stagger parameter (Figure 16), with some amplitude errors but good detection of the shape of the stagger.

It is important to note that the discrete reference measurements obtained with the tCat^®^ device are taken statically, without direct contact with the CW, using vision equipment. In contrast, the results obtained with the monitoring system here presented are dynamic measurements captured during the actual circulation of the train, influenced by the dynamics of the interaction between the pantograph and the OCL. The uplift force of the pantograph with the proposed system, along with the vertical stiffness of the CW, changes the CW height slightly when compared with the steady-state situation. The speed was maintained constant during circulation through the section under study, with a reference value of 45 km/h.

Considering the same KP where there are measurements with the tCat^®^ system, the accuracy of the developed SIA system has been calculated for the CW height. Table 4 lists the minimum, average and maximum errors.

#### 5.2.2. Reproducibility Analysis

After the validation stage, this subsection focuses on assessing the repeatability of measurements obtained by the monitoring system. For this study, two track sections are analysed, namely Section A and Section B, each with a length of 500 m. To evaluate the reproducibility of the measurements, two runs were conducted in each section, both in the same direction and at the same nominal circulation speeds, although in reality, both speeds were not exactly the same.

**Figure 17:**
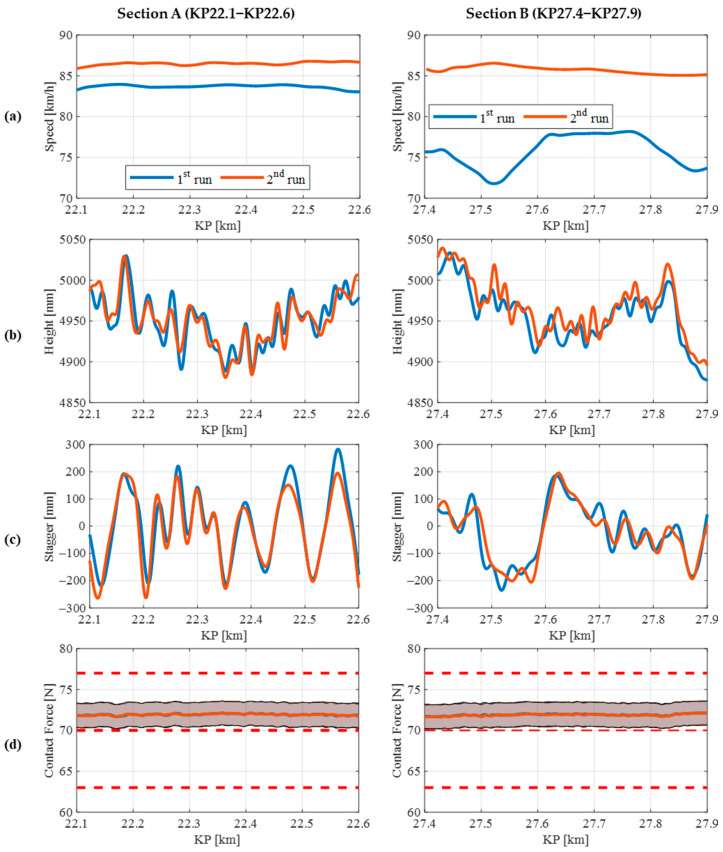
Results of the repeatability performance study of the monitoring system in different Section A (**left**) and Section B (**right**) for four different parameters: speed (**a**), CW height (**b**), CW stagger (**c**) and contact force (**d**).

Figure 17 displays four different parameters (speed (a), CW height (b), CW stagger (c), and estimated contact force (d)) for each section under examination, comparing the two runs. The graph indicates that the measured speed values in Section A are similar between runs, whereas the differences in speed are bigger in the second case. Regarding CW height and stagger, both sections yield highly comparable results in both runs, particularly in the stagger KPI. Lastly, the lower section of the figure showcases the predicted contact force between the pantograph and the CW, displaying the estimated force along with the 95% confidence prediction intervals (represented as shadowed areas). These intervals are required because viscous and dry phenomena in joints are neglected in the calculation. Marques et al. [36] made a revision to the modelling and analysis of friction. It is evident that the estimated force remains consistent in both cases and falls well within the indicated red limits.

Considering the same KP where there are measurements for the first run, the accuracy of the developed SIA system has been calculated for the CW height. Table 5 lists the minimum, average and maximum errors.

## 6. Discussion

**Figure 18:**
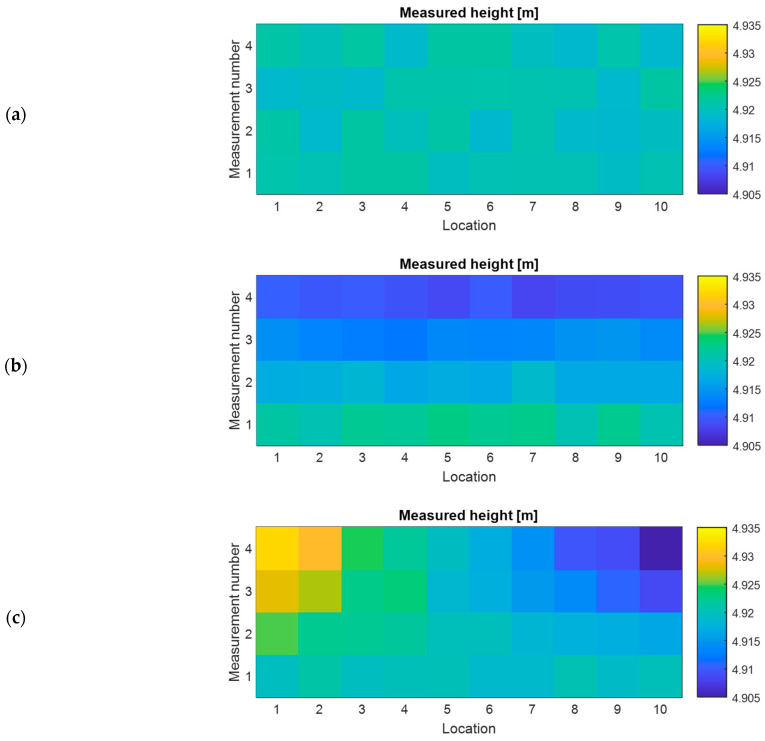
Degradation trend of the CW height. (**a**) Correct height, (**b**) vertical drop and (**c**) incorrect vertical slope.

The monitoring of the OCL provides valuable information about the health status of this crucial asset in the railway system. This monitoring can be conducted continuously or periodically, serving different purposes. Continuous monitoring ensures that the analysed parameters remain consistent, and any deviations could indicate a fault in the system. In such cases, an alert can be sent to the infrastructure manager, enabling prompt action based on the reliability of the detected fault. On the other hand, periodic monitoring is sufficient for assessing the degradation trend over time. Figure 18 shows how a two-dimensional plot can effectively distinguish different failures in the height of the CW. Regions with the same measurement values are represented by flat-coloured surfaces. On the other hand, a continuous vertical drop of the CW shows a colour gradient for different measurements. In the case of an incorrect vertical slope, due, for example, to a broken dropper, the colour gradient intensifies with each measurement.

**Figure 19:**
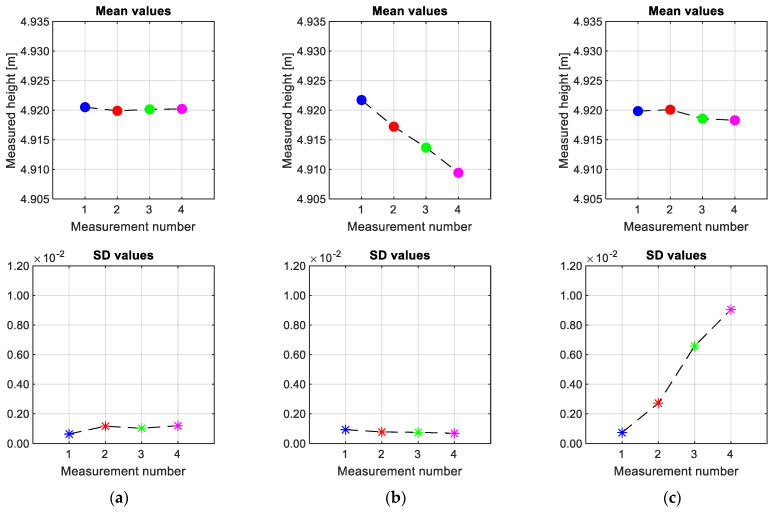
Statistical analysis of the CW height changes. (**a**) Correct height, (**b**) vertical drop and (**c**) incorrect vertical slope.

In addition, conducting a statistical analysis of the measurements recorded at various positions along a track section and at different times allows for a quantitative understanding of the type of failure occurring. Figure 19 shows the mean and standard deviation (SD) values for three different states. In cases where the CW height is consistently correct, both the mean and SD values remain stable, albeit with slight variations between measurements due to the use of a monitoring device. On the contrary, when a failure is present, one of the parameters exhibits stability while the other shows a significant change. A vertical drop in the CW height leads to a shift in the mean value, while an incorrect vertical slope is characterised by a changing SD value. This quantitative method is essential for infrastructure digitalisation platforms to automatically show the locations of required interventions and the corresponding maintenance tasks throughout the entire rail line. As the monitoring system is not as accurate as other costly inspection methods, some differences can occur between measurements. However, assessing the trends of the statistical analysis confirms the feasibility of utilising low-cost devices for maintenance purposes.

The static contact force is a parameter that can be directly obtained from the characterisation of the pantograph. It provides valuable information by quantifying the contact forces at different kilometric points along the railway line. Monitoring the evolution of this contact force can serve as an indicator of contact anomalies and potential issues [37]. It is worth noting that changes in force can be attributed to both the pantograph and the OCL, and using multiple instrumented pantographs can help detect and analyse these changes effectively. In addition to the static contact force, the dynamic contact force is also a valuable parameter for maintenance purposes. Models that describe the interaction between the pantograph and the OCL, such as those employed for stagger characterization using accelerometers, can be utilized to estimate dynamic forces. Machine learning techniques can be applied to further enhance the estimation of dynamic forces, although this area requires further development and research.

The stagger amplitude and the stagger central position, obtained from accelerations of both sides of the pantograph strips, show good repeatability. The reliability of these measurements is influenced by the running conditions of the train unit. In our study, we observed that the measurements exhibit consistent results across different runs and operating conditions, indicating a high level of reproducibility.

By analysing the measured data, the system can promptly detect changes in forces or OCL geometry and alert maintenance personnel in real time. This enables timely adjustments to the pantograph’s force settings to prevent potential interaction problems and mitigate the risk of further failures in the OCL system.

## 7. Conclusions

In this research work, a non-intrusive monitoring system has been developed for the continuous monitoring of overhead contact lines that is adequate to be installed on a train pantograph. The proposed system utilises low-cost sensors and makes use of a set of formulae based on geometrical and mechanical parameters to ensure reliable measurements. The reliability of measurements is further enhanced through physical modelling, which allows for periodic updates of the pantograph’s configuration parameters.

The system modelling is based on a mechanical design approach. The kinematic assessment of the pantograph structure and mechanism is accurately modelled, and the employed models are validated through test-rig experiments. As a result, the position of the pantograph head can be determined based on parameters such as the inclination of the lower arm or push bar. Additionally, by utilising an inclinometer and knowing the height of the carbody roof, the height of the overhead contact line can be monitored.

The static contact force can also be calculated using the inclinometer if the position of the screws controlling the applied torque mechanism is known. Although the calibration of this mechanism is not carried out on a daily basis and the contact force may change over time, variations in the force correspond to changes in the height of a specific pantograph. By employing multiple instrumented pantographs on the same overhead contact line, it is possible to detect whether the change in the pantograph height, and consequently the contact wire height, is due to the stress on the messenger wire or other factors.

The sensors chosen for the monitoring system have been carefully selected thanks to the modelling and laboratory tests conducted on the pantograph. The nature and location of these sensors are well suited to meet the required performance of the monitoring system, given the low-cost and non-intrusive nature of the application.

The field tests conducted have demonstrated the effectiveness of the monitoring system. The results obtained from the monitoring system were found to be comparable to those produced by a commercial device, indicating a high level of accuracy. Furthermore, the obtained results have exhibited repeatability across several runs conducted at different speeds. This consistency in performance and the reliable measurement of the target parameters highlight the robustness of the system and its suitability for real-world applications. The field tests provide confidence in the reliability and accuracy of the monitoring system, positioning it as a viable solution for measuring and monitoring the overhead contact line in operational environments.

## Figures and Tables

**Table 1:** Parameters of the articulated quadrangle.

Parameter	Value	Units
a	1.615	m
b	0.235	m
c	1.400	m
d	0.4643	m
φ	0.5814	rad

**Table 2:** Experimental vertical vibration modes of a pantograph contact strip.

Vibration Mode	Frequency (Hz)
Rigid body	16.79
1st bending mode	57.78
2nd bending mode	133.49

**Table 3:** Comparison of provided results of CW monitoring with previous literature work and existing commercial systems.

			Results of the CW Monitoring			
Ref	Type	Methods	Wear	Tension	Stagger	Height
Xu et al. [27]	On-board	Mechanics	Yes	No	No	No
Derosa et al. [28]	Wayside	Mechanics	No	Yes	No	No
Aydin et al. [29]	On-board	Vision	No	No	Yes	No
Chen et al. [30]	On-board	Vision	No	No	Yes	No
* tcat^®^ [31]	Trolley	Vision	No	No	Yes	Yes
* Catenary Eye [32]	On-board	Vision	Yes	No	Yes	Yes
* Autocommute [33]	On-board	Vision	Yes	No	Yes	Yes
* CAT-T [34]	Trolley	Vision	No	No	Yes	Yes
* CAT-VW [35]	On-board	Vision	Yes	No	Yes	Yes
This work	On-board	Mechanics	No	No	Yes	Yes

* Commercial devices.

**Table 4:** Comparison of error on measured CW height with SIA system against tCAT^®^.

	Absolute Error	Relative Error
Minimum	0.704 mm	0.014%
Average	11.951 mm	0.245%
Maximum	28.980 mm	0.608%

**Table 5:** Comparison of error on measured CW height with SIA system.

	Absolute Error	Relative Error
Minimum	0.010 mm	0.001%
Average	12.503 mm	0.252%
Maximum	42.051 mm	0.847%

## Data Availability

The data are not publicly available due to confidentiality.
